# Effect of *Vipera ammodytes ammodytes* Snake Venom on the Human Cytokine Network

**DOI:** 10.3390/toxins10070259

**Published:** 2018-06-25

**Authors:** Francisc Boda, Krisztina Banfai, Kitti Garai, Augustin Curticapean, Lavinia Berta, Emese Sipos, Krisztian Kvell

**Affiliations:** 1 Department of Fundamental Pharmaceutical Sciences, Faculty of Pharmacy, University of Medicine and Pharmacy of Tirgu Mures, Gheorghe Marinescu Street No. 38, 540139 Tirgu Mures, Romania; boda.francisc@umftgm.ro (F.B.); augustin.curticapean@umftgm.ro (A.C.); 2 Department of Pharmaceutical Biotechnology, Faculty of Pharmacy, University of Pecs, Rokus Street No. 2, 7624 Pecs, Hungary; krisztina.banfai@aok.pte.hu (K.B.); garai.kitti91@gmail.com (K.G.); kvell.krisztian@pte.hu (K.K.); 3 Szentagothai Research Center, University of Pecs, Ifjusag Street No. 20, 7624 Pecs, Hungary; 4 Department of Specialty Pharmaceutical Sciences, Faculty of Pharmacy, University of Medicine and Pharmacy of Tirgu Mures, Gheorghe Marinescu Street No. 38, 540139 Tirgu Mures, Romania; emese.sipos@umftgm.ro

**Keywords:** snake venoms, *Vipera ammodytes*, inflammation-related genes, inflammatory mediators, cytokines, RT-qPCR

## Abstract

Local inflammation is a well-known symptom of envenomation by snakes of the family *Viperidae*, attributed primarily to the phospholipase A_2_s, metalloproteinases and L-amino acid oxidases contained in their venom. The inflammatory effect of snake venoms has been associated with a marked increase of the cytokines IL-1β, IL-6, IL-8, IL-10 and TNF-α. To determine the impact of *Vipera ammodytes ammodytes* snake venom on the expression of inflammation-related genes, we incubated human U937 monocyte cells with dilutions of snake venom. Gene expression was quantified for 28 different genes using a TaqMan^®^ Array Human Cytokine Network 96-well Plate in a RT-qPCR system. Our results have demonstrated that 1.0 μg/mL *Vipera ammodytes ammodytes* venom solution induces a notable change in the expression of several cytokine network genes. Among the upregulated genes, there were several that encode interleukins, interferons, and tumor necrosis factors. We further report the downregulation of three interleukin-related genes. Our findings come as supportive information for the known complex effect of snake venoms on the human cytokine network. It also provides relevant new information regarding the expression of genes that have not been previously associated with the effect of snake venoms.

## 1. Introduction

The inflammatory process represents a defense mechanism of the body against harmful pathogens, damaged cells, or irritating substances. Inflammation can take an acute or chronic form. In its acute form, five typical signs of inflammation are usually present: heat, pain, redness, swelling, and loss of function of the affected tissues or organs. Chronic inflammatory processes are characterized by a continuous and simultaneous destruction and healing of affected tissues, which may lead to chronic inflammatory diseases with detrimental effects on health [1,2].

Following the initiation of acute inflammation, the affected tissues present an increased blood flow and increased permeability of blood vessels; monocytes extravasate to the affected regions and are transformed into macrophages. Macrophages are responsible for antigen presentation and phagocytosis and modulate the immune response through induction of cytokine, chemokine, and growth factor production [1,3]. Macrophages form different subsets depending on the activating signals. Lipopolysaccharides (LPS), interferon gamma (IFN-γ), or interleukin 1 beta (IL-1β) activate M1-type macrophages. These macrophages are known to generate reactive oxygen species (ROS) and to produce proinflammatory cytokines, such as interleukin 6 (IL-6), interleukin 12 (IL-12), IFN-γ, and tumor necrosis factor alpha (TNF-α) [4]. M2-type macrophages are activated by interleukin 4 (IL-4), interleukin 10 (IL-10), or interleukin 13 (IL-13). These macrophages participate in the halting of inflammatory processes and promote tissue recovery through the production of anti-inflammatory mediators, including IL-10, transforming growth factor beta (TGF-β), and IL-1 receptor antagonists [5].

Envenomation by snakes of the family *Viperidae* is associated with both local and systemic effects. Local effects include severe tissue damage, necrosis, hemorrhage, and inflammation of the affected area. Systemic effects are related mainly to the action of snake venom proteins on the cardiovascular system and hemostasis [6,7]. The inflammatory response caused by *Viperidae* snake venoms is attributed primarily to phospholipase A_2_s (svPLA_2_s), snake venom metalloproteinases (SVMPs), and L-amino acid oxidases (LAAOs) contained in the venom. Furthermore, hyaluronidases, nucleases, nucleotidases, phosphomonoesterases, and some nonenzymatic toxins found in snake venoms contribute to the inflammation-inducing effect [8].

Snake venom PLA_2_s are proteins that hydrolyze phospholipids at the sn-2 position, generating lysophospholipids and free fatty acids, including arachidonic acid [9,10]. Arachidonic acid functions as substrate for the synthesis of various proinflammatory mediators, such as leukotrienes (LT), thromboxane A_2_ (TXA_2_), prostacyclin, and prostaglandins (PG). Thus, the inflammatory effect of svPLA_2_s can be directly linked to their enzymatic activity [11,12,13]. However, there are reports of catalytically inactive svPLA_2_s capable of inducing inflammation and nociceptive responses, suggesting the existence of other inflammation-inducing mechanisms not related to the arachidonic pathway [14,15,16].

Snake venom metalloproteinases (SVMPs) are zinc-dependent enzymes responsible for the hemorrhagic, necrotic, and inflammatory effects of snake venoms [17]. Local effects frequently associated with SVMP administration include edema formation, hyperalgesia, leukocyte infiltration, and mast cell degranulation [7,8,18]. SVMP treatment has been associated with the release of various inflammatory mediators, such as IL-1β, IL-6, IL-10, prostaglandin E2 (PGE_2_), and TNF-α [12,19,20].

L-amino acid oxidases (LAAOs) are flavoproteins that catalyze the oxidative deamination of L-amino acids to α-keto acids. The oxidative process leads to the formation of hydrogen peroxide and ammonia. The release of hydrogen peroxide is linked to the apoptotic, cytotoxic, hemorrhagic, and edema inducing effect of LAAOs [8,21]. Furthermore, recent studies have demonstrated that administration of purified LAAOs induces the release of several proinflammatory mediators, such as IL-6, IL-8, TNF-α, PGE_2_, and leukotriene B4 (LTB_4_) [22,23,24].

European venomous snakes are representatives of the genera *Vipera*. Among this genera, the most venomous species is the *Vipera ammodytes* (nose-horned viper) with its two subspecies, *V. ammodytes ammodytes* and *V. ammodytes meridionalis* [25]. This species is widespread in southern Europe, from central and northern Italy to southern Austria, through the Balkans and southern Romania to north-eastern Turkey and southern Caucasia [26]. The most frequently recorded effects of envenomation by *V. ammodytes* are local tissues damage, local and systemic hemorrhage, and to a smaller extent, neurotoxicity [27]. These effects may lead to permanent sequelae and organ function loss and in severe cases, envenomation might have lethal outcomes. Although the frequency of envenomation by *V. ammodytes* recorded in Europe is less than that of envenomation caused by other species in tropical countries, these cases still represent a public health concern, mainly in the Balkan countries [27,28]. While there are several studies focused at isolating and characterizing proteins from the venom of *V. ammodytes* [25,29,30,31,32], reports are scarce on the effects of unfractionated venom—a complex mixture of biologically active proteins—in humans.

Our study aimed to determine the effect of *Vipera ammodytes ammodytes* venom (*VaaV*) on the human cytokine network. Using a TaqMan^®^ Array Plate quantified by RT-qPCR, we assessed the expression of 28 cytokine-associated genes in monocytes treated with *VaaV*.

## 2. Results and Discussion

### 2.1. Effect of Treatment

Viability of cells treated with *VaaV* solution was assessed by microscopic evaluation. *VaaV* caused cell death at concentrations of 3.0, 10, 30, and 100 μg/mL. Monocyte cells treated with 1.0 μg/mL *VaaV* solution showed differentiation towards macrophage lineage as suggested by adherent polygonal cellular shape and growth arrest (Supplementary Figure S1). Cells incubated without treatment were assessed as viable and lacking signs of differentiation (Supplementary Figure S2). Total RNA was isolated from cell cultures deemed viable, namely those treated with 1.0 μg/mL *VaaV* and untreated cells.

### 2.2. Gene Expression in U937 Cells Treated with VaaV

**Figure 1:**
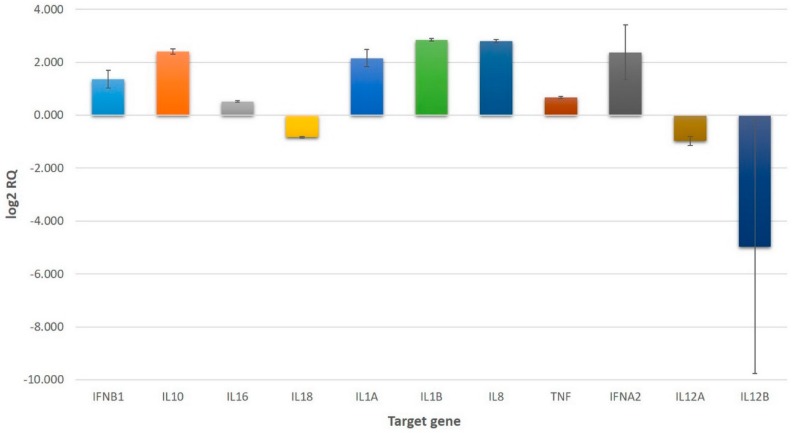
Upregulated and downregulated genes in U937 cells treated with 1.0 μg/mL *VaaV* solution. Values represent mean relative quantification (RQ) error bars represent the standard error on a log2 RQ-based scale. Untreated U937 cells served as reference and are represented by the zero value. The +1 and −1 values represent a two-fold increase or decrease threshold in gene expression.

Gene expression was assayed in triplicate using a TaqMan^®^ Array Human Cytokine Network Plate containing 28 genes coding inflammatory mediators and four endogenous control genes. The endogenous control genes (*18S*, *GAPDH*, *HPRT1*, *GUSB*) allowed for the correction of potential variations in RNA loading. Based on the obtained data, mean fold changes and standard errors were calculated if at least two relative quantification (RQ) values could be measured for one individual gene. The mean fold change of genes following treatment of cells with 1.0 μg/mL *VaaV* solution is presented in Figure 1 in a log2 RQ-based scale. A complete list of genes, obtained RQ values, calculated mean RQs, standard errors, and 90% confidence intervals are presented in Supplementary Table S1. 

#### 2.2.1. Upregulation of Interleukin-Related Genes

Our results showed that *IL1A* and *IL1B* genes presented a significant upregulation, with a mean fold change of 4.67 and 7.21, respectively. These genes encode two members of the IL-1 family: interleukin 1 alpha (IL-1α) and beta (IL-1β). The IL-1 family of cytokines has a major role in the initiation and regulation of inflammation. These cytokines possess a pronounced proinflammatory effect and are capable of inducing the expression of several other cytokines and chemokines, including IL-8 [33,34]. Our findings partially correlate with data available in the literature, as increased levels of IL-1β have frequently been reported following administration of snake venoms [35,36], svPLA_2_s [13,37], or SVMPs [12,20]. However, the expression of IL-1α following envenomation with snake venoms has not yet been the focus of research. Our finding that its gene is similarly upregulated as that of IL-1β suggests that IL-1α might have a pivotal role in the inflammatory process.

IL-10 is an immunosuppressive and anti-inflammatory cytokine that regulates and restrains the inflammatory response by limiting the production of cytokines and chemokines in macrophages and dendritic cells as well as by downregulating the expression of several chemokine receptors [34,38]. Increased IL-10 concentrations have been detected in human patients following envenomation with *Daboia russelii* venom [39] as well as in mice after administration of *Crotalus durissus terrificus* [40] and *Bothrops spp.* venom [41]. Furthermore, several studies have found that administration of SVMPs and svPLA_2_s isolated from *Bothrops* species leads to a marked increase in IL-10 expression [12,18,19]. The expression of IL-10 provides evidence that snake venoms are capable of modulating the expression of both pro- and anti-inflammatory cytokines. In accordance with these results, we report a significant upregulation of the *IL10* gene (5.32-fold) following treatment with *VaaV*. 

We found an approximately 1.4-fold increase in the expression of *IL16*, the gene encoding interleukin 16 (IL-16). IL-16 is a proinflammatory cytokine that functions as a chemoattractant for CD4^+^ and CD8^+^ T cells [34,42]. The expression level of *IL16* in our study is a noteworthy finding, considering the increase of IL-16 levels has not been associated with the effects of snake venoms.

#### 2.2.2. Downregulation of Interleukin-Related Genes

Among the studied genes, we observed the downregulation of two interleukin-encoding genes—*IL18* and *IL12A*—responsible for the expression of interleukin 18 (IL-18) and interleukin 12 subunit alpha (IL-12α), respectively. Although statistically not significant, the results also show an indicative trend of downregulation for *IL12B*, the gene responsible for the expression of interleukin 12 subunit beta (IL-12β). 

We identified a few cases of increased expression of IL-12 following administration of snake venoms or its components [43,44,45] but did not find any reports regarding the expression of IL-18. As both IL-12 and IL-18 induce the production of IFN-γ [34], the downregulation of the genes encoding these cytokines supports our findings regarding the lack of expression of *IFNG*, the gene responsible for encoding IFN-γ.

#### 2.2.3. Upregulation of Chemokine-Related Genes

A marked upregulation following treatment of U937 cells with *VaaV* was observed for *IL8* (6.97-fold increase), the gene encoding interleukin 8 (IL-8). The marked increase in *IL8* expression suggests that IL-8 might be a significant mediator of inflammatory processes induced by snake venom.

IL-8, or C-X-C motif chemokine ligand 8 (CXCL8), is a member of the CXC chemokine family. Its main function involves the recruitment of neutrophils to the site of injury or infection but also functions as a potent chemoattractant for other cell types, including basophils, eosinophils, NK cells, and T cells [34,46]. Release of IL-8 from neutrophils has been reported following in vitro treatment of human neutrophils with *Bothrops bilineata* venom [47] and Cr-LAAO, an L-amino acid oxidase isolated from *Calloselasma rhodosthoma* [22].

#### 2.2.4. Upregulation of Interferon-Related Genes

Interferon alpha (IFN-α) and interferon beta (IFN-β) are members of a highly related protein group called type I interferons (IFN-I). The main function of IFN-I consists of the induction of antiviral responses in cells through different mechanisms [48]. One of these mechanisms involves the direct activation of CD4^+^ and CD8^+^ T cells and dendritic cells and the subsequent release of various cytokines [49,50].

Our results showed a marked increase in the interferon-related gene *IFNB1* (2.7-fold), the gene responsible for the expression of IFN-β. Although statistically not significant, the results also show an indicative trend of upregulation for *IFNA2* (6.57-fold), the gene responsible for the expression of a variant of IFN-α. To the best of our knowledge, we are the first to report the upregulation of interferon-related genes in connection with snake venoms that may support the antiviral activity of certain snake venom components, as suggested by previous reports in other contexts [51,52].

#### 2.2.5. Upregulation of Tumor Necrosis Factor-Related Genes

The tumor necrosis factor superfamily represents a group of cytokines that play an important role in inflammatory processes, immunity and cell proliferation, differentiation and apoptosis, and the formation of secondary lymphoid organs. Tumor necrosis factor alpha (TNF-α) is secreted by macrophages in the acute phase of an inflammation, while lymphotoxin alpha (LT-α, TNF-β) is produced by activated type 1 T helper (Th1) lymphocytes. Both have a pronounced proinflammatory effect and have a significant role in cell apoptosis and tissue necrosis [53,54,55]. The latter is also involved in peripheral lymphoid organogenesis [56].

We found that *TNF*, the gene encoding TNF-α, showed an approximately 1.6-fold increase following the treatment of cells with *VaaV*. The increase in *TNF* expression was to be expected, considering high levels of TNF-α have been observed in numerous experiments involving snake venoms. These experiments used either crude snake venoms [11,35,36,40,43] or isolated snake venom proteins, including SVMPs [19,20,57], svPLA_2_s [13,37,44], or LAAOs [23,58].

### 2.3. Limitations of the Study

The study design does not allow for the differentiation between the primary effect on gene expression induced directly by *VaaV* and the secondary effect on gene expression caused by the cytokines released following the action of *VaaV* treatment.

Furthermore, the use of unfractionated *VaaV* venom in the study does not allow the determination of the contribution to the overall observed effect by individual components contained in the venom. However, the current study design can be easily adapted to measure the effect of individual proteins on gene expression.

## 3. Conclusions

We report the influence of *Vipera ammodytes ammodytes* venom on the expression of a large number of inflammation-related genes in monocytes/macrophages. Various authors have reported the increased expression of IL-1β, IL-6, IL-8, IL-10, and TNF-α cytokines as a consequence of administration of snake venoms or the components thereof. We determined that the genes related to these cytokines, except the gene encoding IL-6, were markedly upregulated in our experiment. Thus, our findings come as supportive information for previous observations. Furthermore, we identified other upregulated genes, namely *IL1A*, *IL16*, *IFNA2*, and *IFNB1*. To the best of our knowledge, the cytokines encoded by these genes have not been previously associated with the effect of snake venoms or their components. Additionally, we report the downregulation of several interleukin-related genes, namely *IL12A*, *IL12B*, and *IL18*. Better understanding of the mechanisms and mediators involved in the inflammatory response following envenomation with snake venoms could be of potential use in the development of targeted venom antiserums.

## 4. Materials and Methods

### 4.1. Snake Venom

Lyophilized *Vipera ammodytes ammodytes* venom was obtained from the Institute of Immunology, Zagreb, Croatia. A stock solution with a concentration of 10 mg/mL was prepared by dissolving the lyophilized *VaaV* in phosphate buffered saline (PBS) (Lonza, Basel, Switzerland).

### 4.2. Cell Line

U937 cell line was purchased from the American Type Culture Collection (ATCC CRL-1593.2™) and cultured in RPMI-1640 medium (Lonza, Basel, Switzerland) supplemented with 5 mL L-glutamine (Lonza, Switzerland), 10 mL penicillin/streptomycin (Lonza, Basel, Switzerland), and 50 mL fetal bovine serum (FBS) (EuroClone, Milan, Italy). Cell cultures were maintained in a 5% CO_2_ atmosphere at 37 °C for 72 h. Cell viability before treatment was assessed using Olympus CKX41 microscope (Olympus, Tokyo, Japan).

### 4.3. Treatment of Cells

U937 cell cultures were each treated with 1.0, 3.0, 10, 30, and 100 μg/mL *VaaV* solution for 48 h. Cells incubated without treatment were used as negative control. Following incubation, cell viability was assessed microscopically. Cells deemed viable were collected in RA1 lysis buffer (Macherey-Nagel GmbG, Düren, Germany) and stored at −80 °C until further analysis.

### 4.4. RNA Isolation and cDNA Construction

To determine the expression of genes associated with the human cytokine network, total RNA was isolated from cell cultures treated with 1.0 μg/mL *VaaV* solution and untreated cell cultures (negative control). Total RNA was isolated using a NucleoSpin RNA II kit (Macherey-Nagel, Düren, Germany) based on the manufacturer’s recommended protocol. The obtained RNA concentrations were determined using a Nanodrop 2000 spectrophotometer (Thermo Fischer Scientific, Waltham, MA, USA). The isolated RNA was reverse transcribed to cDNA with a PikoReal 96 Real-Time PCR System (Thermo Scientific, Waltham, MA, USA) using a High-Capacity cDNA Reverse Transcription Kit (Applied Biosystems, Foster City, CA, USA) according to the manufacturer’s instructions. The PCR program used for cDNA synthesis consisted of sample incubation for 2 min at 50 °C and 10 min at 95 °C, followed by 40 cycles at 95 °C for 15 s and 60 °C for 60 s.

### 4.5. RT-qPCR

Gene expression was determined using a TaqMan^®^ Array Human Cytokine Network 96-well Plate (Part No. 4414255, Applied Biosystems, USA). The TaqMan^®^ Array Plate contains 28 assays for genes associated with pro- and anti-inflammatory cytokines and four assays for candidate endogenous control genes.

The quantitative real-time PCR amplification was performed on a 7500 Real-Time PCR System (Applied Biosystems, USA) in a 20 μL volume containing TaqMan Universal PCR Master Mix (Applied Biosystems, USA) and the cDNA samples. The PCR program used consisted of sample incubation for 2 min at 50 °C and 10 min at 95 °C, followed by 40 cycles at 95 °C for 15 s and 60 °C for 1 min. All assays were plated in triplicate. The obtained amplification data was evaluated using the 7500 Software v2.0.6 (Applied Biosystems, USA). Calculations and statistical analysis of the results was performed using Graphpad Prism software (version 6.0), GraphPad Software, La Jolla, CA, USA).

## Supplementary Materials

The following are available online at http://www.mdpi.com/2072-6651/10/7/259/s1, Figure S1: Monocyte cells treated with 1.0 μg/mL *VaaV* solution showing differentiation towards macrophage lineage as suggested by adherent polygonal cellular shape and growth arrest. Figure S2: Viable monocytes, lacking signs of differentiation following incubation without treatment, serving as negative control. Table S1: RQ values measured following treatment of U937 cells with 1.0 μg/mL *VaaV*. All assays were plated in triplicate. Untreated cells served as reference (negative control). Mean RQ, standard error, and 90% confidence interval (using t-distribution for small set of samples) were calculated if at least two values were measured.
