# Supplementary material for: Effect of Vipera ammodytes ammodytes Snake Venom on the Human Cytokine Network
Source: Toxins (Basel). 2018 Jun 25;10(7):259. doi: 10.3390/toxins10070259 (PMC6070926; doi:10.3390/toxins10070259)
Supplement: Supplementary file 1 [file toxins-10-00259-s001.pdf]

Supplementary Materials: Effect of *Vipera ammodytes ammodytes* Snake Venom on the Human Cytokine Network

Francisc Boda, Krisztina Banfai, Kitti Garai, Augustin Curticapean, Lavinia Berta, Emese Sipos and Krisztian Kvell

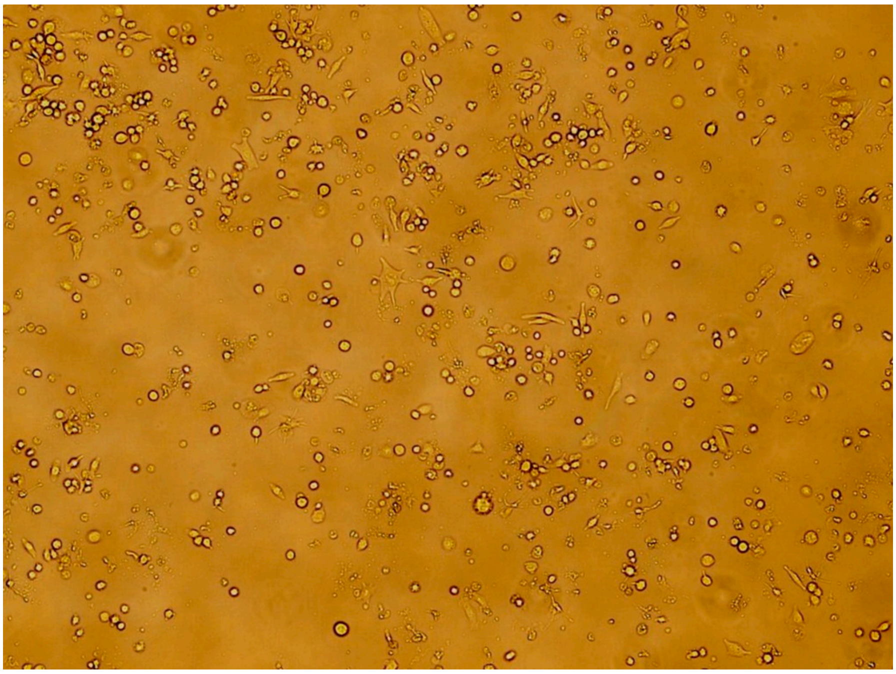

Figure S1. Monocyte cells treated with 1.0 μg/mL *VaaV* solution showing differentiation towards macrophage lineage as suggested by adherent polygonal cellular shape and growth arrest.

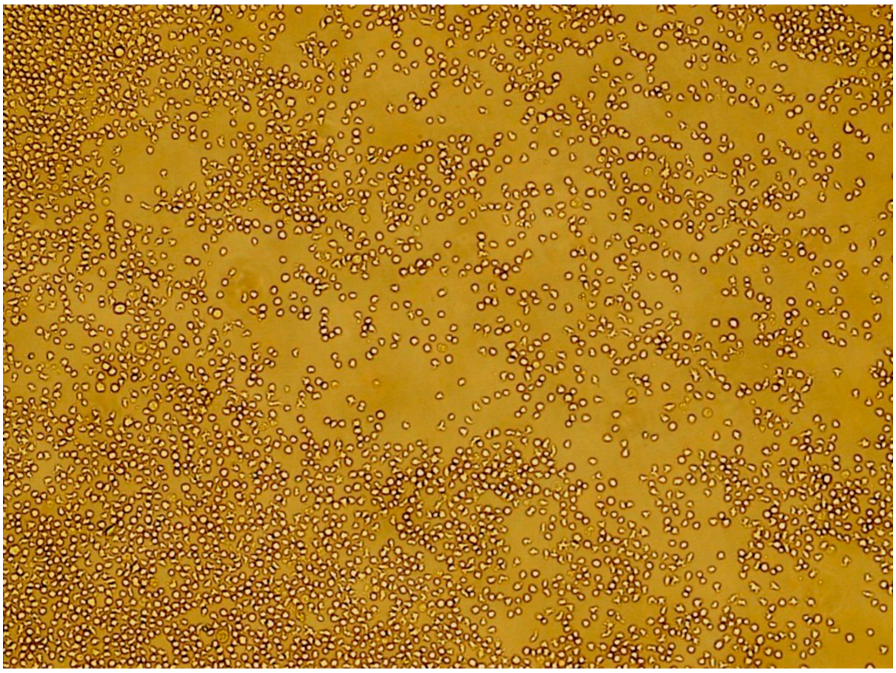

Figure S2. Viable monocytes, lacking signs of differentiation following incubation without treatment, serving as negative control.

Table S1. Relative quantification (RQ) values measured following treatment of U937 cells with 1.0 µg/mL *VaaV*. All assays were plated in triplicate. Untreated cells served as reference (negative control). Mean RQ, standard error, and 90% confidence interval (using t-distribution for small set of samples) were calculated if at least two values were measured.

**Target name**	**Sample (1 µg/mL *VaaV*)**			
	Measured RQ (n=3)	Mean RQ	SE	CI (90%)
18S [1]	ND<br>ND<br>ND	NI	NI	NI
GAPDH [1]	ND<br>ND<br>ND	NI	NI	NI
HPRT1 [1]	ND<br>ND<br>ND	NI	NI	NI
GUSB [1]	ND<br>ND<br>ND	NI	NI	NI
IFNA1	ND<br>ND<br>ND	NI	NI	NI
IFNA16	ND<br>ND<br>ND	NI	NI	NI
IFNA17	ND<br>ND<br>ND	NI	NI	NI
IFNA2	2.525<br>ND<br>10.620	6.572	4.048	6.572 ± 25,556
IFNA6	ND<br>ND<br>ND	NI	NI	NI
IFNA7	5.964<br>ND<br>ND	NI	NI	NI
IFNA8	1.914<br>ND<br>0.665	NI	NI	NI
IFNB1	2.788<br>3.654<br>1.658	2.700	0.578	2.700 ± 1,686
IFNG	ND<br>ND<br>ND	NI	NI	NI
IL10	5.841<br>4.700	5.321	0.333	5.321 ± 0,972

	5.422			
	0.454			
IL12A	0.572	0.513	0.059	0.513 ± 0,374
	ND			
	0.886			
IL12B	0.001	0.444	0.443	0.444 ± 2,794
	ND			
	ND			
IL13	ND	NI	NI	NI
	ND			
	ND			
IL15	ND	NI	NI	NI
	ND			
	1.435			
IL16	1.490	1.436	0.031	1.436 ± 0.089
	1.384			
	ND			
IL17A	ND	NI	NI	NI
	ND			
	0.569			
IL18	0.575	0.561	0.011	0.561 ± 0,033
	0.538			
	6.062			
IL1A	5.094	4.672	1.642	4.672 ± 2,767
	2.861			
	7.134			
IL1B	6.859	7.213	0.231	7.213 ± 0,673
	7.646			
	ND			
IL2	ND	NI	NI	NI
	ND			
	ND			
IL3	ND	NI	NI	NI
	ND			
	ND			
IL4	ND	NI	NI	NI
	ND			
	9.880			
IL5	ND	NI	NI	NI
	ND			
	1.411			
IL6	0.457	NI	NI	NI
	ND			
	7.540			
IL8	6.493	6.969	0.306	6.969 ± 0,893
	6.875			
IL9	ND	NI	NI	NI

	ND			
	ND			
	2.919			
LTA	ND	NI	NI	NI
	ND			
	1.596			
TNF	1.512	1.589	0.043	1.589 ± 0,124
	1.659			

[1] Endogenous control genes. ND: Not detectable. NI: Not interpretable. SE: Standard error; CI: Confidence interval; *GAPDH*: glyceraldehyde-3-Phosphate Dehydrogenase ; *HPRT1*: hypoxanthine phosphoribosyltransferase 1; *GUSB*: glucuronidase beta; *IFNA*: interferon alpha; *IFNB*: interferon beta; *IFNG*: interferon gamma; *IL*: interleukin; *LTA*: lymphotoxin alpha; *TNF*: tumor necrosis factor.
